# Increased serum IL-36β and IL-36γ levels in patients with neuromyelitis optica spectrum disorders: association with disease activity

**DOI:** 10.1186/s12883-019-1415-2

**Published:** 2019-08-05

**Authors:** Chun-Sheng Yang, Qiu xia Zhang, Yu Deng, Bing jie Zhou, Lin jie Zhang, Li min Li, Yuan Qi, Jing Wang, Li Yang, Fu-Dong Shi

**Affiliations:** 1 Department of Neurology, Tianjin Neurological Institute, Tianjin Medical University General Hospital, No 154 Anshan Road, Heping District, Tianjin, 300052 China; 2 Department of Neurology, Tianjin TEDA Hospital, No 65 The Third Road, Tianjin Economic Technological Development Area, Tianjin, 300457 China; 3 Department of Neurology, Barrow Neurological Institute, St. Joseph’s Hospital and Medical Center, Phoenix, AZ 85013 USA

**Keywords:** Neuromyelitis optica spectrum disorders, Interleukin 36, Biomarkers

## Abstract

**Background:**

Interleukin 36 (IL-36) cytokines belong to the IL-1 family and play an important role in some autoimmune diseases. However, the relationship between IL-36 and neuromyelitis optica spectrum disorders (NMOSD) remains unclear.

**Methods:**

We determined serum IL-36α, IL-36β and IL-36γ levels and assessed correlations with clinical characteristics in 50 NMOSD patients and 30 healthy controls (HC).

**Results:**

The concentrations of serum IL-36β and IL-36γ were significantly higher in patients with NMOSD than in HCs and decreased during remission. Serum IL-36β levels were positively correlated with the annual relapse rate (ARR), spinal cord lesion length and Expanded Disability Status Scale (EDSS) scores.

**Conclusions:**

Serum IL-36β and IL-36γ levels were related to disease activity in NMOSD patients and may be important biomarkers of NMOSD.

## Background

Neuromyelitis optica spectrum disorders (NMOSD) are severe inflammatory disorders of the central nervous system (CNS), which preferentially affect the optic nerve and spinal cord. NMOSD comprise six core clinical characteristics: optic neuritis (ON), acute myelitis (AM), area postrema syndrome, acute brainstem syndrome, acute diencephalic clinical syndrome and symptomatic cerebral syndrome [1]. The discovery of immunoglobulin G autoantibodies specific for aquaporin-4 (AQP4-IgG) was critical for understanding the pathology of NMOSD [2, 3]. AQP4-IgG enters the CNS through the damaged blood-brain barrier (BBB), binds to AQP4 on astrocyte endfeet, and activates the complement system, which induces astrocyte damage, granulocyte infiltration, oligodendrocyte death, and neuronal cell death [4].

In addition to AQP4-IgG, cytokines/chemokines, such as IL-6, IL-17, IL-10, IL-12, interferon gamma (IFN-γ), B-cell activating factor (BAFF), CXCL13 and tumour necrosis factor alpha (TNF-α), may also be involved in the pathogenesis of NMOSD [5–7]. However, the role of newly recognized members of the IL-1 family, such as IL-36, remains unclear. The IL-36 cytokines include 3 agonists (IL-36α, IL-36β, and IL-36γ) and 1 antagonist (IL-36Ra); the agonists bind specifically to a common receptor composed of IL-36R and subsequently form a heterodimer with the IL-1R accessory protein (IL-1RAcP) to stimulate inflammation [8]. IL-36 cytokines are mainly expressed by keratinocytes, the bronchial epithelium, neuronal cells, glial cells, dendritic cells and macrophages. Recent studies have demonstrated that IL-36 is a pro-inflammatory cytokine in several autoimmune diseases, including systemic lupus erythematosus (SLE), multiple sclerosis (MS), inflammatory bowel disease, dermatitis, psoriasis, acne and hidradenitis suppurativa [9–15]. However, the possible role of IL-36 in NMOSD is unknown. Therefore, in this study, we determined the serum IL-36 levels of patients with NMOSD and explored the potential relationship between IL-36 and clinical parameters.

## Methods

### Patients

Patients with NMOSD (*n* = 50) who were admitted to Tianjin Medical University General Hospital, Tianjin, China, from December 2015 to December 2018 were recruited. Patients with NMOSD were diagnosed according to the 2015 international consensus diagnostic criteria for NMOSD [1]. Patients who had a history of connective tissue disorders or other immunoinflammatory diseases were excluded. We did not include myelin oligodendrocyte glycoprotein immunoglobulin G (MOG-IgG)-positive patients because the pathophysiology of MOG-IgG-associated NMOSD is probably different from that of AQP4-IgG-positive NMOSD [1]. In the acute phase, all patients with NMOSD were treated with high-dose intravenous methylprednisolone (IVMP) (0.5 g/day for 3–5 consecutive days) and then maintained with low-dose corticosteroids and/or appropriate doses of immunosuppressive agents during remission. In addition, we enrolled 30 healthy age- and sex-matched individuals from the Health Care Center of our hospital.

This study was approved by the ethics committee of Tianjin Medical University General Hospital, and all participants provided written informed consent prior to participation.

### Data and sample collection

The data for demographic and clinical characteristics, age at onset, annual relapse rate (ARR), spinal cord lesion length, and Expanded Disability Status Scale (EDSS) scores at nadir were acquired from medical records. EDSS scores at nadir were evaluated during acute exacerbations before high-dose IVMP by two neurologists.

Fifty serum samples were obtained during acute exacerbations before administration of high-dose IVMP, and 32 serum samples were collected during remission. As a control, 30 HC serum samples were obtained from the Health Care Center of our hospital. All serum samples obtained from participants were stored at − 80 °C until the time of the assay.

### Detection of anti-AQP4 antibodies

MOG-IgG tests and AQP4-IgG tests were conducted in our clinical neuroimmunological laboratory. As previously described [16], AQP4-IgG was detected by a cell-based assay (CBA).

### Measurement of IL-36 levels

Serum IL-36α, IL-36β and IL-36γ concentrations were evaluated using a human IL-36α, IL-36β and IL-36γ enzyme-linked immunosorbent assay (ELISA) kit (R&D Systems, USA) according to the manufacturer’s instructions. Optical densities were measured at 450 nm, and IL-36α, IL-36β and IL-36γ concentrations were evaluated with reference to a standard curve. The lowest detectable levels of these cytokines were 12.5 pg/ml, 12.5 pg/ml and 18.75 pg/ml, respectively.

### Statistical analyses

Statistical analysis was performed using the Statistical Package for the Social Sciences (SPSS 22.0), and graphs were created using GraphPad Prism 6.01. We applied t-tests or the Mann–Whitney U test for quantitative data and the chi-squared test or Fisher’s exact test for qualitative data. The relationships between IL-36 and clinical parameters were analysed using Spearman’s correlation coefficient. *P*-values < 0.05 were considered statistically significant for all statistical tests.

## Results

### Demographic and clinical characteristics

The demographic and clinical characteristics of 50 NMOSD patients and 30 HCs are summarized in Table 1.

**Table 1:** Demographic and clinical characteristics of NMOSD and HC

	NMOSD(50)	HC (30)	P
Gender, n (% female)	48(98%)	27(90%)	0.358
Age at sampling, years	46.46 ± 15.04	51.30 ± 12.71	0.144
Age at onset, years	41.9 ± 15.71	–	–
Follow-up duration, years	4.66 ± 4.33	–	–
Annualized relapse rate (ARR)	2.24 ± 0.97	–	–
EDSS at nadir	3.98 ± 2.12	–	–
Presentation at sampling, n (%)			
ON	7(14%)	–	–
Area postrema syndrome	2(4%)	–	–
AM	26(52%)	–	–
Brain stem syndrome	6(12%)	–	–
Diencephalic clinical syndrome	3(6%)	–	–
Cerebral syndrome	6(12%)	–	–
length of newly identified spinal cord lesion (vertebral segments)	5(1, 15)	–	–
AQP4-Ab, n (%)	28(56%)	–	–
Imunossupressive agents and dosage, n (%)			
Prednisonlone (12 mg/d)	5(10%)	–	–
Azathioprine (2 mg/kg.d)	8(16%)	–	–
Mycophenolate Mofetil(1.5 g/d)	7(14%)	–	–
Rituximab^a^	30(60%)	–	–

Abbreviations: *NMOSD* neuromyelitis optica spectrum disorders, *HC* healthy control, *ARR* annualized relapse rate, *EDSS* Kurtzke Expanded Disability Status Scale, *ON* optica neuritis, *AM* acute myelitis

^a^: All patients were treated with rituximab (Biogen-Idec, Cambridge,MA, and Genentech, San Francisco, CA) 100 mg (equivalent of 50–59 mg/m^2^) IV, one infusion per week for 3 consecutive weeks. Continued dosage was dependent on the percentage of circulating CD19^+^ B-cell counts. Whenever it reached 1% of total lymphocyte population, rituximab 100 mg was reinfused

### Serum IL-36 levels

**Fig. 1:**
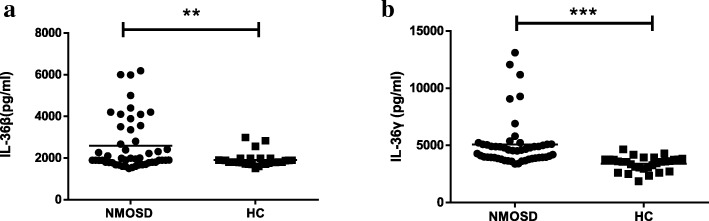
Serum IL-36β and IL-36γ levels in NMOSD patients and HC. NMOSD = neuromyelitis optica spectrum disorders; HC = healthy controls. **a** Comparison of serum IL-36β levels in NMOSD patients and HC. **b** Comparison of serum IL-36γ levels in NMOSD and HC. **P < 0.01, ***P < 0.001

**Fig. 2:**
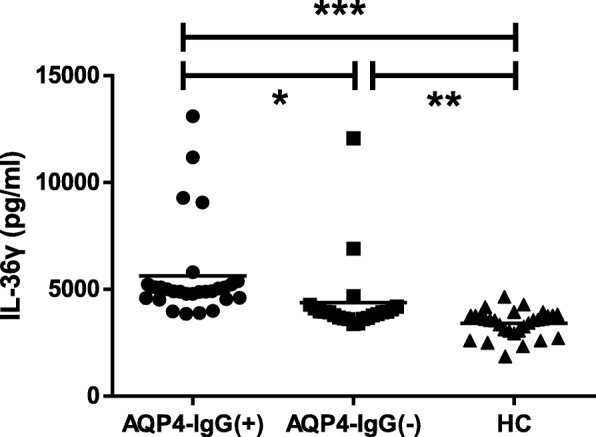
Serum IL-36γ levels among the 30 HC, 28 AQP4-IgG (+) and 22 AQP4-IgG (−) in NMOSD patients. AQP4-IgG (+) = AQP4-IgG-positive; AQP4-IgG (−) = AQP4-IgG-negative. *P < 0.05, **P < 0.01, ***P < 0.001

**Fig. 3:**
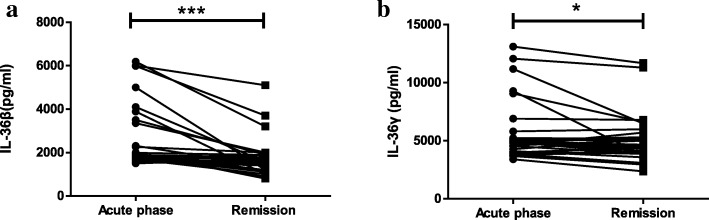
Serum IL-36β and IL-36γ levels in the acute and remission phases in 32 NMOSD patients. **a** Comparison of serum IL-36β levels between acute and remission in NMOSD patients. **b** Comparison of serum IL-36γ levels between acute and remission in NMOSD patients. *P < 0.05, ***P < 0.001

As shown in Fig. 1, serum IL-36β and IL-36γ levels were significantly increased in patients with NMOSD compared to those of HCs (*P* = 0.005, *P* < 0.0001, respectively, Fig. 1a and b), but there was no significant difference in serum IL-36α levels between the two groups (*P* = 0.118, figure not shown). Furthermore, no significant difference in serum IL-36α and IL-36β levels was found between AQP4-IgG-positive and AQP4-IgG-negative NMOSD patients (*P* = 0.379 and 0.141, respectively, figure not shown). Serum IL-36γ levels in AQP4-IgG-negative NMOSD patients were significantly lower than those in AQP4-IgG-positive NMOSD patients (*P* = 0.040, Fig. 2) but significantly higher than those in HCs (*P* = 0.001, Fig. 2). We also found that serum IL-36β and IL-36γ levels were significantly decreased during the remission period compared to those during the acute phase (*P* = 0.0001 and 0.013, respectively, Fig. 3a and b). Pearson correlation results showed time of immunosuppression wasn’t correlated with IL-36β level either in acute or remission phase (r = 0.242, *P* = 0.183; r = − 0.151, *P* = 0.409, figure not shown). Although there was correlation between time of immunosuppression and IL36-γ level in acute phase(r = 0.381, *P* = 0.031, figure not shown), no correlation was found between time of immunosuppression and IL36-γ level in remission phase (r = 0.117, *P* = 0.525, figure not shown). Serum IL-36α levels were lower during the remission period than during the acute phase in NMOSD patients, but this difference did not reach the level of statistical significance (*P* = 0.342, figure not shown).

### Correlations between serum IL-36 levels and clinical parameters

**Fig. 4:**
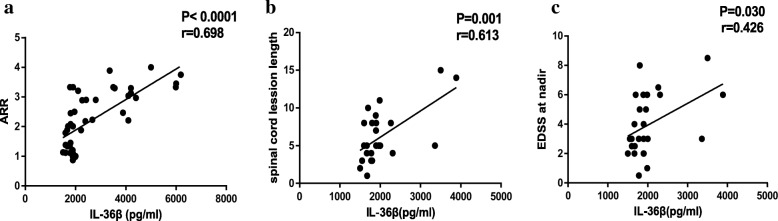
Correlation between serum IL-36β levels and clinical characteristics in NMOSD patients. **a** Correlation between serum IL-36β levels and ARR in 44 NMOSD patients. ARR = annual relapse rate. **b** Correlation between serum IL-36β levels and spinal cord lession length in 26 NMOSD patients. **c** Correlation between serum IL-36β levels and EDSS at nadir in 26 NMOSD patients. EDSS = Expanded Disability Status Scale

We performed a correlation analysis between serum IL-36 levels and clinical parameters to explore potential associations. The relationship between ARR and serum IL-36 levels for 44 NMOSD patients is shown in Fig. 4; the other six NMOSD patients were excluded because the follow-up duration was less than 3 months. A significant positive correlation was observed between serum IL-36β levels and ARR (r = 0.698, *P* < 0.0001, Fig. 4a), while neither serum IL-36α levels nor serum IL-36γ levels were correlated with ARR in patients with NMOSD (r = 0.087, *P* = 0.574; r = 0.136, *P* = 0.378, figure not shown). Considering that the EDSS scores of patients with different clinical syndromes of NMOSD may vary during acute exacerbations, we enrolled 26 NMOSD patients who presented with acute attacks of AM for further study. We found that serum IL-36β levels were positively correlated with the length of newly identified spinal cord lesions and the EDSS scores at nadir in patients with NMOSD patients (r = 0.613, *P* < 0.001, Fig. 4b; r = 0.426, *P* = 0.030, Fig. 4c, respectively). Furthermore, neither serum IL-36α levels nor serum IL-36γ levels correlated with the length of newly identified spinal cord lesions (r = 0.116, *P* = 0.571; r = 0.129, *P* = 0.529, respectively, figure not shown). We found that there were no significant correlations between serum IL-36α levels and EDSS scores (r = − 0.011, *P* = 0.957, figure not shown) or serum IL-36γ levels and EDSS scores (r = 0.005, *P* = 0.980, figure not shown).

## Discussion

Previous studies have reported increased serum IL-36 levels in patients with autoimmune disorders, and serum IL-36 may be involved in the pathological process associated with autoimmune diseases. Emerging data have suggested that IL-36 is a pro-inflammatory signal that triggers further inflammatory mediators and a potential therapeutic target for the treatment of immune diseases in humans [14, 15]. In the present study, we first demonstrated that serum IL-36β and IL-36γ levels were significantly increased in patients with NMOSD compared to those in HC, especially in the acute stage, suggesting that IL-36 might play an important role in the inflammatory pathogenesis of NMOSD. Recent research indicates that serum IL-36α and IL-36γ levels are markedly increased in SLE patients and are strongly correlated with SLE Disease Activity Index (SLEDAI) scores and complement C3 levels [16]. Furthermore, IL-36γ levels are elevated in patients with atopic dermatitis and have pro-inflammatory effects on human endothelial cells [10, 17]. In addition, a recent report showed that IL-36α and IL-36γ may play a pro-inflammatory role in the pathophysiology of inflammatory bowel disease [9]. The present study showed that serum IL-36β and IL-36γ levels were significantly decreased during the remission period compared to those in the acute phase. Serum IL-36β levels were positively correlated with ARR, the length of newly identified spinal cord lesions and the EDSS scores at nadir. IL-36β is associated with the severity of disease in patients with NMOSD. These findings indicated that IL-36 was associated with disease activity of NMOSD patients. Further studies are required to investigate the possible role of IL − 36 in NMOSD. Despite an increasing number of studies on the pathophysiology and signalling pathways associated with IL-36 during the immune response, few breakthroughs have been reported. Previous studies suggest that IL-36α, IL-36β and IL-36γ signal through IL-36R and IL-1RAcP to activate a pathway that leads to NF-κB and MAPKs [5, 18, 19]. In addition, IL-36 potently stimulates human M2 macrophages, Langerhans cells and keratinocytes to produce pro-inflammatory cytokines [20]. However, the pathophysiological mechanism and signalling pathway of IL-36 in NMOSD remain unclear.

A previous study showed that IL-36γ is upregulated in models of experimental autoimmune encephalitis (EAE) and that microglia are a potential target of IL-36γ [21]. Furthermore, IL-36γ derived from neutrophils could stimulate microglia to produce neutrophil-stimulating cytokines [21]. This finding was consistent with those studies, which showed that IL-36γ induced both the production of such cytokines in other myeloid cells residing outside the CNS and the recruitment of neutrophils [19, 22–24]. Neutrophils play a prominent role in the pathogenesis of NMOSD because abnormal neutrophil aggregation was found in NMOSD lesions [3, 25], and neutrophil protease inhibition reduces AQP4-IgG damage in the mouse brain [26]. Yang reported increased plasma levels of epithelial neutrophil-activating (ENA) peptide 78/CXCL5 during the remission of NMOSD [27]. That study showed that ENA 78 plasma levels correlated positively with EDSS scores in NMOSD patients. The overproduction of pro-inflammatory cytokines, such as IL-1β activates, ENA 78, which in turn leads to neutrophil infiltration into lesions. All these findings suggest that IL-36γ could contribute to neuroinflammation, perhaps by promoting neutrophil recruitment.

Rafael reported that the IL-36 receptor was expressed by human blood and intestinal T lymphocytes and was activated via IL-36β in a dose-dependent manner, inducing proliferation of CD4+ T lymphocytes [28]. The direct induction of IL-17A by IL-36 agonists was observed in cultured murine CD4+ T cells [22]. A study also showed that Th17 cytokines, such as IL-17A, directly induced IL-36 cytokines and in turn enhanced their own expression and the production of pro-inflammatory cytokines, such as IL-6 and IL-8, in cultured human keratinocytes, forming a positive feedback loop between IL-36 and Th17 cytokines [29]. Many studies have also shown increased CSF IL-6 and IL-17 levels in patients with NMO [30–32]. Th17- and Th2-related cytokines are upregulated in the CSF of NMO patients [31]. All of these results indicated that IL-36 could play an important role in the pathogenesis of NMOSD.

This study had certain limitations. First, as it is retrospective, bias is inevitable. Second, the patients were insufficient in number without a control group with another CNS autoimmune disease, for example MS. In the future, we will recruit more NMOSD patients in a perspective study with a control group with another CNS autoimmune disease, which may lead to a deeper understanding of the role of IL-36 in NMOSD.

## Conclusions

The present study first showed that serum IL-36β and IL-36γ levels were increased in NMOSD patients, especially during the acute stage. Serum IL-36β levels were positively correlated with ARR, spinal cord lesion length and EDSS scores. Due to the small sample and the observational nature of this study, additional research on larger samples is needed to investigate pathological mechanisms and signalling pathways related to IL-36 in NMOSD.

## Data Availability

The datasets used and/or analyzed during the current study are available from the corresponding author on reasonable request.

## Abbreviations

AM: Acute myelitis
AQP4-IgG: Immunoglobulin G autoantibodies specific for aquaporin-4
ARR: Annual relapse rate
BAFF: B-cell activating factor
BBB: Blood-brain barrier
CBA: Cell-based assay
CNS: Central nervous system
EAE: Experimental autoimmune encephalitis
EDSS: Expanded Disability Status Scale
ELISA: Enzyme-linked immunosorbent assay
ENA: Epithelial neutrophil-activating
HC: Healthy controls
IFN-γ: Interferon gamma
IL: Interleukin
IL-1RacP: IL-1R accessory protein
IVMP: High-dose intravenous methylprednisolone
MOG-IgG: Myelin oligodendrocyte glycoprotein immunoglobulin G
MS: Multiple sclerosis
NMOSD: Neuromyelitis optica spectrum disorders
ON: Optic neuritis
SLE: Systemic lupus erythematosus
SLEDAI: SLE Disease Activity Index
SPSS: Statistical Package for the Social Sciences
TNF-α: Tumour necrosis factor alpha
